# Ephedrine QoS: An Antidote to Slow, Congested, Bufferless NoCs

**DOI:** 10.1155/2014/691865

**Published:** 2014-08-28

**Authors:** Juan Fang, Zhicheng Yao, Xiufeng Sui, Yungang Bao

**Affiliations:** ^1^College of Computer Science, Beijing University of Technology, Beijing 100124, China; ^2^Institute of Computing Technology, Chinese Academy of Science, Beijing 100190, China

## Abstract

Datacenters consolidate diverse applications to improve utilization. However when multiple applications are colocated on such platforms, contention for shared resources like networks-on-chip (NoCs) can degrade the performance of latency-critical online services (high-priority applications). Recently proposed bufferless NoCs (Nychis et al.) have the advantages of requiring less area and power, but they pose challenges in quality-of-service (QoS) support, which usually relies on buffer-based virtual channels (VCs). We propose QBLESS, a QoS-aware bufferless NoC scheme for datacenters. QBLESS consists of two components: a routing mechanism (QBLESS-R) that can substantially reduce flit deflection for high-priority applications and a congestion-control mechanism (QBLESS-CC) that guarantees performance for high-priority applications and improves overall system throughput. We use trace-driven simulation to model a 64-core system, finding that, when compared to BLESS, a previous state-of-the-art bufferless NoC design, QBLESS, improves performance of high-priority applications by an average of 33.2% and reduces network-hops by an average of 42.8%.

## 1. Introduction

Web service companies such as Google, Yahoo, Amazon, and Microsoft deploy datacenters with hundreds to thousands of machines to host millions of users, all of whom may be running large, data-intensive applications [1]. Latency-critical interactive applications must provide quality of service that is predictable and often strictly defined. To satisfy variable daily demands and to avoid contention for shared memory and network resources, datacenter operators overprovision resources, which results in poor resource utilization. Finding better ways to deliver the required QoS is thus essential for improving datacenter efficiency and managing costs.

Networks-on-chip are important shared resources in the manycore devices that will likely be used to build future datacenters. The NoCs in such chips are responsible for conveying operands between cores, accessing main memory, managing coherence, and performing I/O [2–4].

Traditional NoCs use router buffers to reduce the number of dropped or deflected (misrouted) packets. These buffers, however, improve effective bandwidth at the expense of design complexity, chip area, and power consumption [4, 5]. Furthermore, these costs increase with the number of cores, making bufferless NoCs attractive for large-scale manycore chips [5, 6].

In contrast, bufferless NoCs eliminate on-chip router buffers so that when a flit arrives, a router must immediately select an appropriate output port to forward it. Although previous studies show that bufferless NoCs can reduce router area by 60% and save power consumption by 40% [5], the difficulty in providing QoS in such designs has prevented their use in datacenter environments with latency-critical applications. The NoCs used in datacenters generally rely on buffers to create virtual channels for different levels of service [7].

To address the problem, we propose QBLESS, a QoS-aware bufferless NoC scheme targeting datacenters. Instead of using the prevalent VC-based QoS mechanisms, QBLESS tags flits with priority bits and leverages this information in its deflection routing and congestion-control mechanisms. The flits of latency-critical applications are assigned a high priority, making them privileged with respect to routing in the QBLESS NoC.

QBLESS routers implement two arbitration mechanisms based on priority information. First, the routing mechanism always allocates appropriate output ports to privileged flits and deflects flits of low-priority applications. To avoid livelock, the high-priority flits undergo loss of privilege after *N* hops, where *N* is a system parameter influenced by factors such as application memory access characteristics and network size (for more details, see Section 3.1). Second, QBLESS adopts a dynamic source-throttling mechanism to control network congestion according to two rules: (1) privileged sources will never be throttled and (2) the throttling rates of nonprivileged sources are proportional to their IPFs (instructions-per-flit), a measure that indicates memory access intensity.

To enable the QBLESS NoC scheme, we add a QoS-register to each core and design a router architecture that can be programmed for various applications demands. We study QBLESS in simulator, experimental results which show that QBLESS effectively improves QoS and performance. Compared to BLESS [5], a current state-of-the-art bufferless NoC, QBLESS improves the performance of latency-critical applications by up to 55.1% (60.0%) in a 64-core (100-core) system with an 8 × 8 (10 × 10) mesh NoC. Average improvement is 33.2% (38.2%). Somewhat counterintuitively, QBLESS does not hurt low-priority applications but improves their performance by 1.7%, on average, over BLESS.

## 2. Background and Related Work

Datacenters are built from high-end chip multiprocessor (CMP) servers. CMPs rely on efficient networks-on-chip to synchronize cores and to coordinate access to shared memory and I/O resources. Here we present background specific to datacenter NoCs and briefly survey the most relevant prior work.

### 2.1. QoS and Utilization in Datacenters

In datacenters using CMPs with tens of cores, more and more workloads are deployed on a single server, and thus they must share resources. Kambadur et al. [8] point out that in Google datacenters, an average of 14 hyperthreads form heterogeneous applications run simultaneously on one server. For instance, on a single machine, there may be five to even twenty unique applications running together. Such mixed workloads degrade application performance. In particular, they can influence the QoS of interactive online services, which is strongly related to user experience and is a key factor in the revenue of the Internet companies. Datacenter operators thus overprovision resources to guarantee QoS to these latency-critical applications, even if doing so lowers resource utilization. For instance, Google [9] reports that CPU utilization in a typical 20,000-server datacenter for online services averaged about 30% during January through March, 2013. In contrast, batch-workload datacenters averaged 75% CPU utilization during the same period.

Modern datacenters sacrifice server utilization to guarantee the QoS of online services by separating them from batch workloads. Previous efforts to increase utilization while keeping a high level of QoS have colocated the two incompatible kinds of workloads on the same node to eliminate interference. Tang et al. explore the impact of the shared memory subsystem (including the last level cache (LLC) and front side bus (FSB)) on Google datacenter applications [10]. They propose ReQoS [1] to monitor the QoS of latency-sensitive applications and adaptively reduce the memory demands of low-priority applications. They also study the negative effects that nonuniform memory access (NUMA) [11] brings to Google's important web services like the Gmail backend and web-search frontend.

Previous work on guaranteeing datacenter QoS mainly focuses on the on-chip and off-chip memory subsystems. However, just as the security level is defined by the weakest component, QoS is dictated by the least robust participant: this means that all shared resources must be QoS-aware if any are to meet service-level agreements (SLAs). Improving NoC QoS technology for interactive datacenter applications is thus one promising direction for achieving higher throughput and greater energy-efficiency.

### 2.2. QoS-Aware Buffered NoCs

Dally and Towles [7] show that adding buffers to create virtual channels not only prevents deadlock but also makes it possible to provide different levels of service.

Many buffer-based QoS approaches have thus been proposed for NoCs. For example, MANGO [12] guarantees QoS by prioritizing virtual circuits and partitioning virtual channels (VCs) with different priorities. Instead of prioritizing VCs, Bolotin et al. [13] propose prioritizing control packets over data packets. Das et al. [14] propose application-aware prioritization policies to improve overall application throughput and ensure fairness in NoCs. Grot et al. [15] propose a preemptive virtual clock (PVC) scheme to reduce dedicated VCs for QoS-support. Ouyang and Xie [16] design LOFT, a scheme that leverages a local frame-based scheduling mechanism and a flow-control mechanism to guarantee QoS. Grot et al. propose Kilo-NOC [17], a topology-aware QoS NoC architecture, that can substantially reduce buffer overhead.

### 2.3. Bufferless NoCs

Some recent work focuses on alternative designs that are tradeoff power consumption, die area, and performance. One promising direction is bufferless routing [5], which temporarily misrouts or drops and retransmits packets to effectively resolve output port contention. Moscibroda and Mutlu [5] propose the BLESS routing algorithm which consists of a set of rules for routers to select flits and output ports. Fallin et al. [18] propose the CHIPPER router architecture to reduce the complexity of BLESS control logic.

Bufferless routing yields significant network power savings with minimal performance loss when the network load is low-to-medium. In such bufferless NoCs, router area is reduced by 40–75% and power consumption is reduced by 20–40% [5, 6, 18]. However, for network-intensive workloads, bufferless routing behaves much worse than traditional buffered NoCs due to high deflections rates and bandwidth saturation.

To bridge the performance gap between the buffered and bufferless NoCs at high network load, one possible approach is to directly improve the efficiency of bufferless deflection routing. Previous work [6, 19–21] uses source-throttling or constraining applications' network request rates to reduce deflection-rates and improve overall system throughput. Nychis et al. [6] propose the BLESS-throttling (BLESS-T) algorithm to mitigate congestion by limiting traffic from NoC-insensitive applications. Ausavarungnirun et al. [19] propose an application-aware mechanism, adaptive cluster throttling (ACT), to improve throughput and fairness by throttling cluster of application. Kim et al. [20] propose clumsy flow-control (CFC) to degrade network congestion by implementing credit based flow-control in bufferless NoCs.

Another approach is to make a hybrid network that can adaptively switch between the higher-capacity buffered mode and lower-cost bufferless mode. Jafri et al. [22] propose adaptive flow-control (AFC) to allow routers to switch between backpressure mode (in which they store incoming flits) and back pressureless mode (in which they use deflection), which performs well under both high and low network loads.

Previous proposals are effective in improving throughput and fairness of bufferless NoCs but they are not suited for datacenter environments with mixed workloads where the performance of latency-critical applications might be substantially degraded. In this work, we investigate both congestion control and deflection routing, finding that the latter is more effective in guaranteeing QoS in bufferless NoCs.

### 2.4. QoS-Aware Bufferless NoCs

Since almost all QoS-support techniques are based on buffer-based VCs, implementing QoS-support in bufferless NoCs remains an open problem.

**Figure 1:**
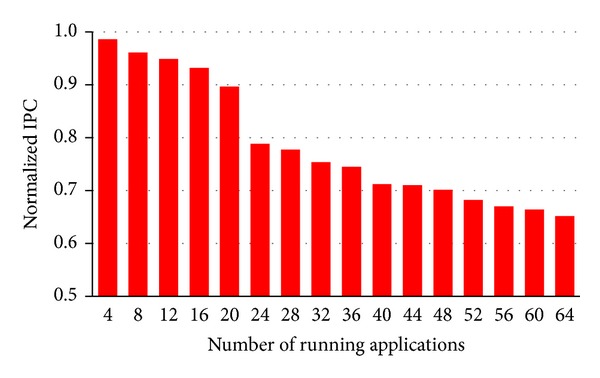
Performance decay of h264ref due to NoC contention (experimental setup is in Section 4).

NoCs are shared by many cores; a QoS-oblivious bufferless NoC may substantially degrade performance for latency-critical applications, even if overall throughput is high. To investigate this, we simulate a 64-core system and measure the impact of NoC contention. We designate h264ref from SPEC CPU2006 [23] to be a high-priority application, and we randomly mix it with other (low priority) applications.  Figure 1 illustrates that, as the number of additional applications increases from three to 63, the IPC (instruction per cycle) of h264ref declines by 35%.

**Figure 2:**
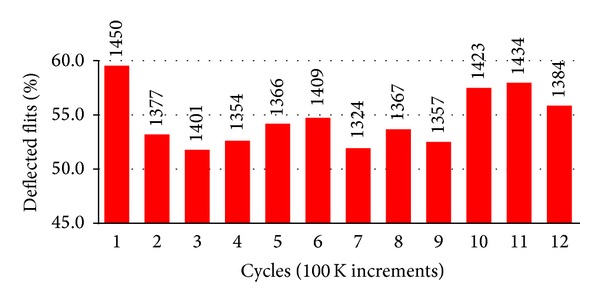
Percentage of deflected flits of mcf w/BLESS-T.

Figure 2 illustrates that BLESS-T, a state-of-the-art bufferless routing and congestion-control mechanism, still performs poorly with respect to guaranteeing SLA-level QoS for datacenter environments (here we take mcf from SPEC CPU2006 as the critical application). There are two reasons for this. First, BLESS-T allows data flits from high-priority critical applications to be deflected by low-priority flits. Figure 2 shows that mcf suffers from severe flit deflection at a rate of 51–59% (54% on average) in an 8 × 8 NoC. Second, since BLESS-T uses IPF as the metric to perform source-throttling, a critical application with low IPF may be chosen as the throttling victim. Thus the application stays in a starvation state in which it is prevented from injecting flits into network. For example, our experimental results show that the throttling rate of mcf is 40%, on average.

On the one hand, bufferless NoCs have the advantages of small area and low power. On the other hand, SLA-level QoS-support is essential for improving datacenter utilization through resource sharing. These factors motivate us to investigate how to design and implement QoS on bufferless NoCs.

## 3. QBLESS Design

**Figure 3:**
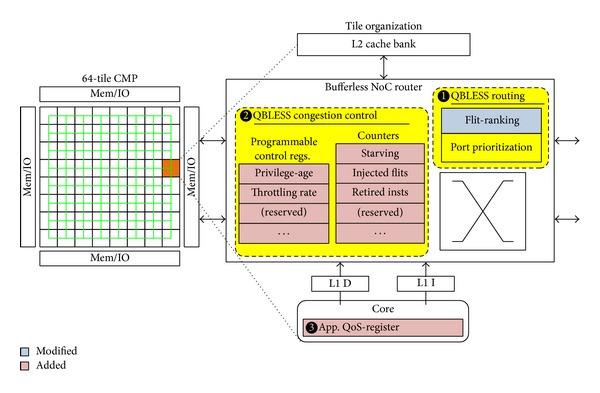
QBLESS router architecture.

We propose QBLESS, a QoS-aware bufferless NoC design, for datacenter environments.  Figure 3 illustrates the organization of our QBLESS scheme. In particular, QBLESS consists of three components: (1) a bufferless routing mechanism is responsible for selecting appropriate output ports for incoming flits in light of priority information (Section 3.1);  (2) a congestion-control mechanism implements source throttling, obeying a new set of rules to adjust throttling rates (Section 3.2); and (3) a tagging mechanism conveys application flit priority information to NoC routers (Section 3.3).

To integrate these mechanisms into NoCs, we need to add a set of registers and to modify some components in the routers.  Figure 3 shows these modules we add (in red) and the modules we modify (in blue). We present the details of our three mechanisms in the following subsections.

### 3.1. QBLESS Routing (QBLESS-R)

#### 3.1.1. Illustrative Example

**Figure 4:**
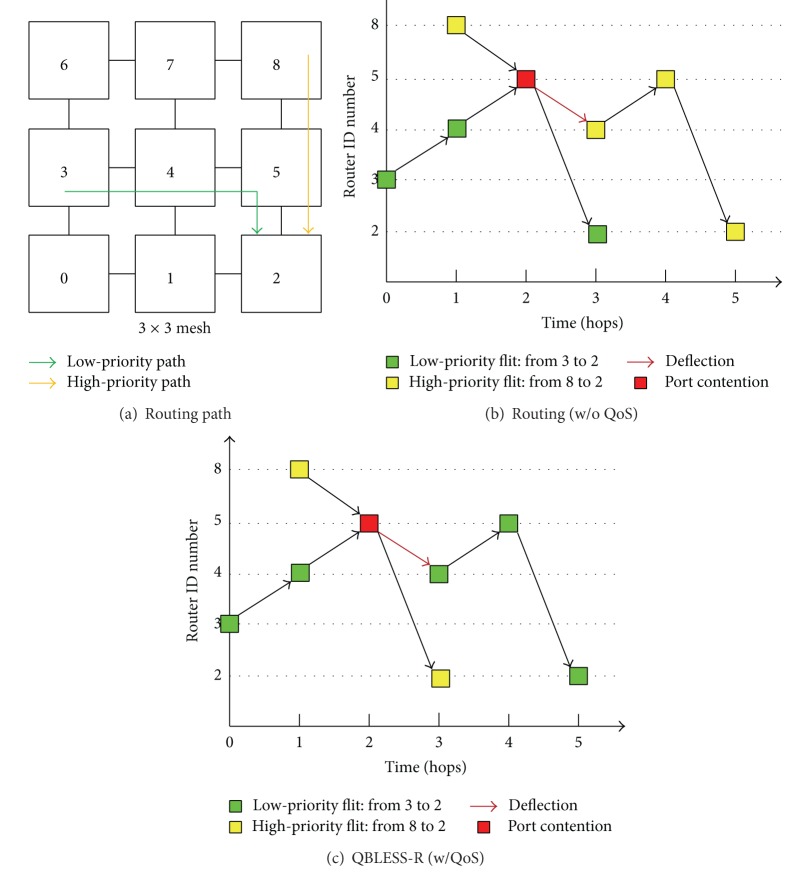
Example of QBLESS-R.

Figure 4 illustrates the principle of the QBLESS-R mechanism. A high-priority application sends a flit via path (8→5→2) according to the rules of dimension-order routing, as shown in Figure 4(a). Meanwhile, a low-priority application wants to send a flit through path (3→4→5→2). The flit of the low-priority application is sent one cycle before its competitor so that the two flits arrive at router number 5 at the same time. They contend for the same output port to router number 2. Since there is no buffer, one must be deflected in a wrong direction.

Previous routing algorithms usually adopt age-based arbitration to determine which flit to deflect, regardless of the priority of the data flits. Therefore, in this case, because the age of the low-priority flit is one hop larger than that of the high-priority flit, the high-priority flit is deflected to router number 4, as shown in Figure 4(b).  Figure 4(c) shows that QBLESS allows the high-priority flit to go through router number 5 and deflects the low-priority flit to router number 4. Thus, compared to the QoS-unaware routing algorithm, QBLESS removes two hops from the path of the high-priority flit.

To achieve this, a QBLESS router must perform two tasks: ranking flits to select an appropriate flit candidate (flit-ranking) and prioritizing available output ports to select an appropriate one (port-prioritizing).

#### 3.1.2. Flit-Ranking

Previous routing algorithms usually adopt age-based arbitration, for example, BLESS using an oldest-first (OF) ranking policy that performs best in most scenarios in terms of latency, deflection-rate, and energy-efficiency [5]. However, the OF-only ranking policy is unaware of priority, which means that flits of latency-critical applications will inevitably be deflected.

In QBLESS, a high-priority flit obtains a* privilege-age* when injected into the NoC, which means that the age of the high-priority flit is a certain number of hops ahead of low-priority flits. As shown in Figure 3, the value of this* privilege-age* is stored in a register and is programmable via software.

Determining the value of the* privilege-age* is critical to effectiveness of the QBLESS routing. The value should be large enough to allow high-priority applications to always beat low-priority ones. For low-priority applications,* privilege-age* should be small enough to avoid livelock.

The value of* privilege-age* is determined by the network parameters (size, diameter), the individual application characteristics (IPF and data locality), and the network confliction. In practice,* privilege-age* is an empirical parameter that reflects QBLESS's ability to guarantee high-priority applications. Since it is programmable, we can dynamically adjust its value according to NoC performance. We evaluate the impact of* privilege-age* in Section 5.2.

#### 3.1.3. Port-Prioritizing

When a flit arrives at the router, the router first tries to assign it to its preferred port. If the preferred port is occupied, the router assigns it to a deflecting port. The routing algorithm must guarantee that low-priority flits are not deflected indefinitely.

### 3.2. QBLESS Congestion Control

Starvation occurs due to congestion when a router exhausts all its ports and cannot inject new flits into the NoC. For bufferless NoCs, congestion increases the deflection-rate, and deflections exacerbate congestion. Thus, congestion control is important for both throughput and latency.

Throttling a specific application is an effective approach for mitigating starvation, but it degrades the performance of the victim application. Previous studies [6, 19–21] try to enforce overall system throughput and fairness according to IPF, MPKI (misses per kilo-instructions), and injection rate. Although such mechanisms (e.g., BLESS-T) are effective for improving fairness, they are unsuitable for datacenter environments.

**Figure 5:**
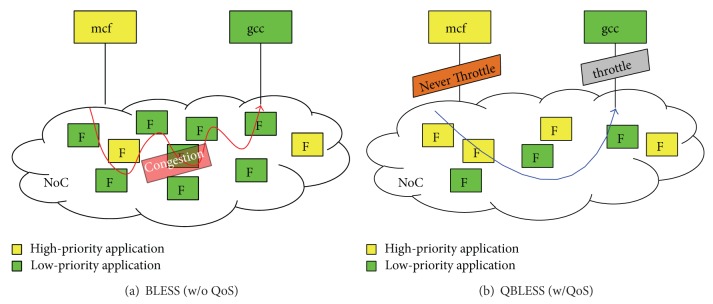
Example of QBLESS-CC.

Figure 5 illustrates the principles of the QBLESS-CC mechanism. Like previous schemes, QBLESS-CC also adopts source-throttling to control congestion. In contrast to those previous schemes, QBLESS-CC can recognize network nodes injecting high-priority flits and avoid throttling them, as shown in Figure 5(b).

Table 1 illustrates QBLESS-CC rules. Program execution is divided into a series of epochs. During each epoch, each network node performs two tasks: determining throttling rate and monitoring/updating statistics. To achieve these goals, we add a set of registers to each router to record the dynamic throttling rate and to track the number of starvation cycles, injected flits, and retired instructions (see Figure 3). There is a global controller that periodically collects these data to identify congestion spots and to calculate throttling rates. Specifically, QBLESS-CC needs to address the following three issues.

#### 3.2.1. When to Throttle

In each epoch, a global controller collects the IPF and starvation rate of each router. If any router's starvation rate exceeds a threshold, the network is deemed to be congested. Note that each router has its own threshold:

(1) $$\begin{array}{c} \text{threshold}=\min\left(\alpha +\frac{\beta }{\text{IPF}+\text{priority}\times \lambda },\gamma \right). \end{array}$$

Equation (1) defines the relationship between IPF, priority, and threshold. These coefficients are not fixed and can be changed by the operating system.

#### 3.2.2. Whom to Throttle

Generally, high-priority applications are not targeted for throttling. Low-priority applications whose IPFs are lower than the average value are selected as throttling candidates.

#### 3.2.3. How Much to Throttle

Lower IPF indicates less NoC sensitivity, and thus applications with lower IPF can be throttled more than others. In particular, we adopt the algorithm of BLESS-T [6] and add priority to the calculation of the throttling rate as shown in (2). As in (1), all coefficients are programmable:

(2) $$\begin{array}{c} {\text{throttle}}_{\text{rate}}=\min\left(\rho +\frac{\sigma }{\text{IPF}+\text{Priority}\times \varphi },\tau \right). \end{array}$$

### 3.3. QoS Identification

Each flit has a (potentially multibit) priority tag. For example, one bit can be used to indicate two priority levels. To make the QBLESS scheme easy to understand, we use just one bit to present our design. In practice, priority levels can be extended (Section 5.3).

The* priority tags* are obtained from application QoS registers in the CPU cores. Specifically, we leverage a QoS framework that adds priority information to each process control block (PCB), and we add a corresponding QoS-register to each core (see Figure 3). The priority information is programmed by the operation system (OS). Upon a context switch, the OS stores the value of the QoS-register into the PCB of the old process and then loads the new priority value from the process to be run. On each memory access request, the core reads the value from the QoS-register and sets the priority value for the request. Thus all the NoC packets contain this priority information.

## 4. Methodology

### 4.1. Simulator Model

We use MacSim [24], a trace-driven, cycle-level, heterogeneous architecture simulator. MacSim models a detailed pipeline (in-order and out-of-order), a memory system that includes caches, the NoC, and the memory controllers. We model an 8 × 8  (10 × 10)-mesh CMP.  Table 2 shows the parameters of our system. We run 10 million cycles for each experiment.

### 4.2. Workloads

We evaluate randomly generated multiprogrammed workloads from 29 SPEC CPU2006 on both the 64-core and 100-core systems. Each workload consists of one high-priority application and other low-priority applications. For each application, we capture the instruction trace of a representative execution slice using a Pin tool [25].

### 4.3. QBLESS Parameters

We determine the following algorithm parameters based on empirical evaluations [6]:* privilege-age *is set to 32, and the period of network information collection *T* is set to 100 K cycles. For the congestion threshold, we set the range limit from *α* = 0.01 to *γ* = 0.7. We set the coefficient *β* = 0.4 and the priority associated factor *λ* = 2. We set the throttling rate interval to go from *ρ* = 0.25 to *τ* = 0.8, and the factor *σ* = 0.9, *φ* = 2.

### 4.4. Comparison Mechanisms

To evaluate QBLESS, we implement two previously proposed bufferless routing and congestion-control mechanisms in our simulator: BLESS [5] and BLESS-T [6].

## 5. Evaluation

In this section, we evaluate the effectiveness and scalability of QBLESS.

### 5.1. Overall Performance

#### 5.1.1. High-Priority Applications

**Figure 6:**
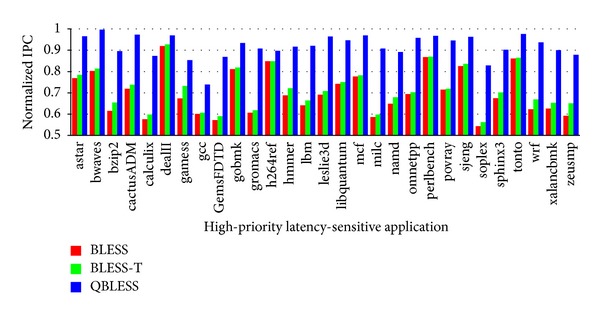
Performance of high-priority applications.

Figure 6 shows the IPC slowdown of the high-priority applications in each of the 29 workloads. The results are normalized to solo execution (the selected high-priority application runs alone). According to Figure 6, QBLESS reduces IPCs by less than 10% on average. This is much better than BLESS, which is unaware of SLA-level QoS. Although BLESS-T can reduce network congestion to improve system performance by throttling network nodes, it is unable to distinguish high-priority applications from low-priority ones. High-priority applications thus suffer from being heavily throttled. As expected, our results demonstrate that, for high-priority applications, QBLESS performs much better than methods that have no SLA-level QoS guarantee mechanism.

#### 5.1.2. Low-Priority Applications

**Figure 7:**
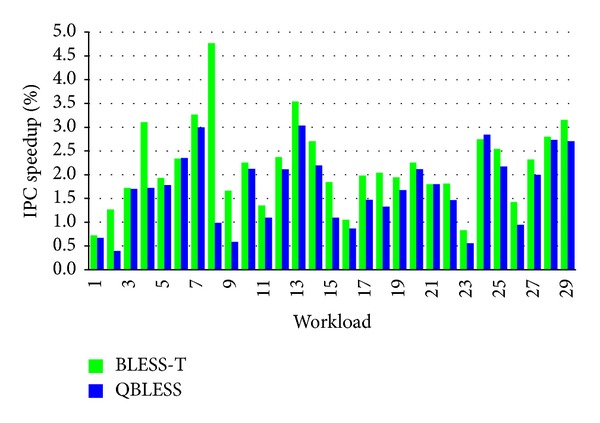
Performance of low-priority applications (normalized to BLESS).

Although QBLESS can guarantee the QoS requirements of high-priority applications, the overall throughput of other low-priority applications is also improved.  Figure 7 illustrates these counterintuitive results; QBLESS improves the throughput of low-priority applications by 0.4%~3.0% and 1.7% on average. Compared to BLESS-T, the overall system throughput of the most low-priority workloads drops negligibly by only 0.4%.

There are two reasons for this: first, QBLESS-CC can reduce network congestion to improve overall system performance; second, QBLESS-R ensures that the flits of low-priority applications arrive at their destinations after a certain number of hops of delay.

Based on Figures 6 and 7, we conclude that QBLESS improves performance for high-priority applications with negligible impact on corunning low-priority applications.

### 5.2. Analysis

#### 5.2.1. Performance Breakdown of Routing and Throttling

**Figure 8:**
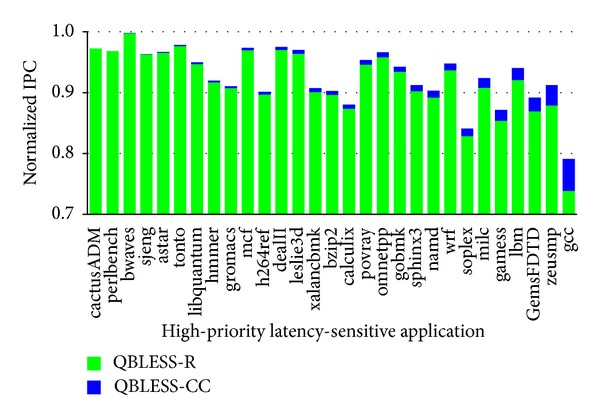
Performance breakdown of routing and congestion control (normalized to solo).

Figure 8 illustrates the performance breakdown of the routing mechanism and the congestion-control mechanism. The bars are sorted in the ascending order of QBLESS-CC. Figure 8 shows that QBLESS-R contributes more than 90% to the performance improvement of the high-priority applications, indicating the effectiveness of QBLESS-R.

On the other hand, congestion control is also effective for some applications, such as gcc, although the benefit is not that obvious due to relative low network intensity of our workload traces. In fact, as pointed out by Nychis et al. [6], network congestion can cause application throughput reductions for both small and large network loads. So we believe that QBLESS can gain more benefits from QBLESS-CC when network is more heavily congested.

#### 5.2.2. Average Network Hops

**Figure 9:**
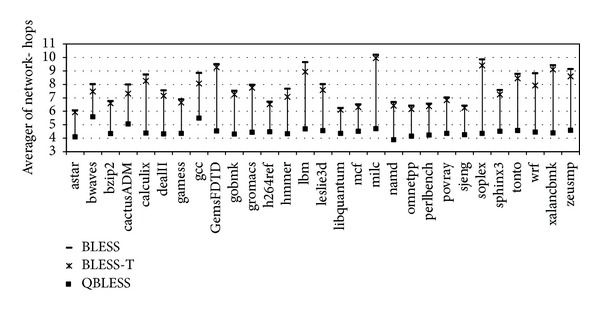
Average network-hops of high-priority applications.

As illustrated in Figure 9, the network-hops of QBLESS are 3.9 to 5.6 (4.4 on average), which reduces the average network-hops by 41.7% and 38.9%, respectively, compared to BLESS and BLESS-T. The deflection-rate of high-priority application is largely reduced, since QBLESS-R prioritizes the flits of latency-critical application and assigns the preferred ports.

#### 5.2.3. Privilege-Age

**Figure 10:**
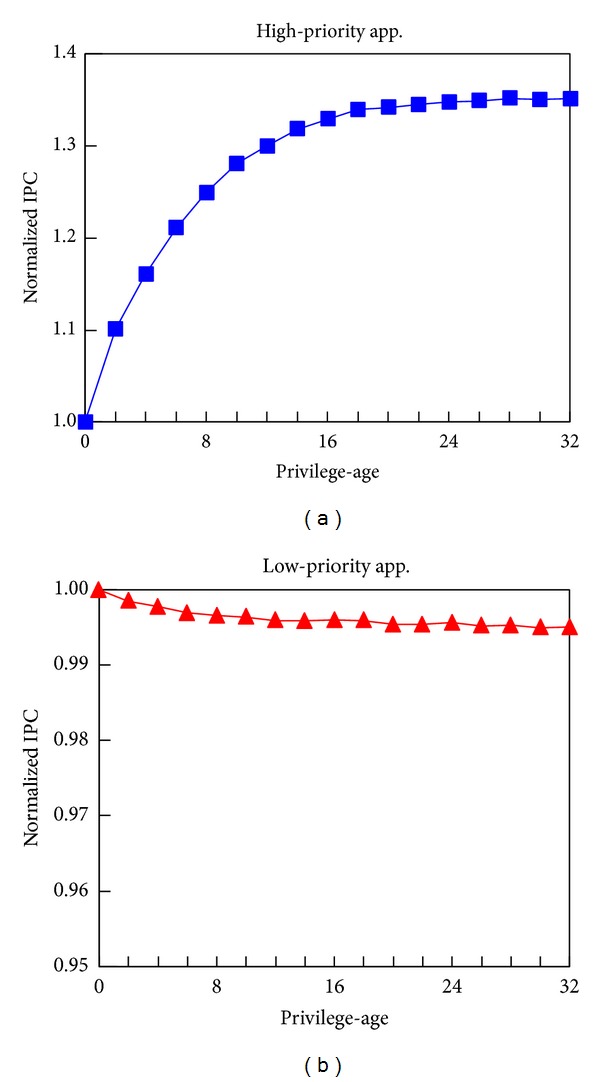
The impact of privilege-age.

As mentioned in Section 3.1, the value of* privilege-age* is critical to the effectiveness of QBLESS-R but is difficult to be determined. We conduct many experiments and results in Figure 10 show that 32 is a good enough empirical value for* privilege-age*. It is interesting that* privilege-age* has negligible impact on low-priority applications. Therefore, we choose* privilege-age* = 32 for QBLESS evaluation. It is worth noting that* privilege-age* is programmable.

### 5.3. Scalability

#### 5.3.1. Multiple Priorities

**Figure 11:**
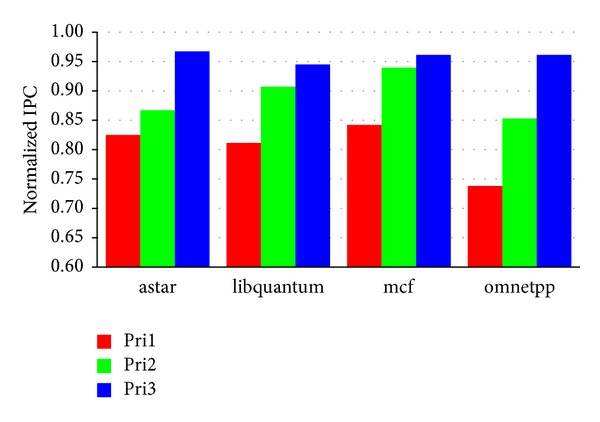
The performance impact of different priorities.

In previous experiments, QBLESS supports only two priorities. We extend QBLESS to support 4 priorities (three-level high priorities and one low priority) by using two priority bits.  Figure 11 shows that higher priority yields better performance. For example, the highest priority applications achieve 94.5%~96.7% performance compared to solo while the middle and lower priority applications achieve 85.3%~90.7% and 73.8%~84.2%, respectively. These gradient performance results conclude that QBLESS can be extended to support multipriority easily with very low cost.

#### 5.3.2. 100 Cores

**Figure 12:**
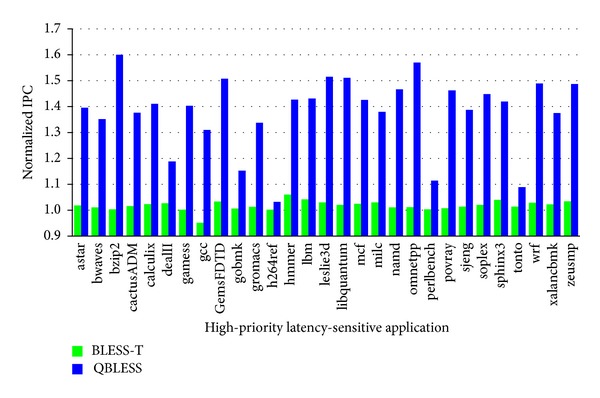
Performance of high-priority applications (100 cores, normalized to BLESS).

We perform experiments to evaluate QBLESS in a 100-core system. As shown in Figure 12, compared to BLESS and BLESS-T, QBLESS improves the performance of latency-critical application by 3.2%~60.0% (38.2% on average) and 3.0%~59.4% (35.7% on average), respectively, which is more significant than the 64-core system. This means that QBLESS can achieve good performance scalability with SLA-QoS as the number of core increases.

### 5.4. Hardware Overhead

The major source of hardware overhead of QBLESS is the modification of router architecture, which is required to measure the starvation rate at each node and to throttle injection. As shown in Figure 3, in each router, QBLESS requires three 32-bit counters and two 8-bit control registers. Additionally, an 8-bit register is required in each core to store the QoS information derived from the application level. Each tile, containing one process core and one router, requires only 15 bytes (=3 × 4B + 2 × 1B + 1B) of storage overhead in total, which is much less than the storage overhead for implementing the buffered router (256 bytes per router).

## 6. Conclusion

We propose QBLESS, a hardware programmable approach for reducing in-network contention in bufferless NoCs. QBLESS adaptively selects the routed output port and throttling rate of low-priority applications to ensure the QoS of high-priority latency-critical corunners. We examine both application level and network level performance in 8 × 8 and 10 × 10 networks and show significant QoS improvements for latency-critical applications on a variety of real workloads.

## Figures and Tables

**Table 1:** Interval-based QBLESS congestion control.

Each node	Global controller
(1) Dynamically throttle according to global controller information from previous quantum (2) Monitor IPF and starvation throughout this quantum	(1) Collect node measurements from previous quantum (2) Identify congestion spots (3) Calculate throttling rate for next quantum (4) Broadcast throttling rate (5) Wait for next quantum and repeat

**Table 2:** System parameters for evaluation.

Network	Topology	2D mesh, 8 × 8 (10 × 10) size
	Routing algorithm	QBLESS (BLESS)
	Routing latency	2 cycles
Core	Out-of-order, 16 MSHR, 128 instructions window size	
	L1 I-cache and D-cache: 32 KB, 64 B line-size, 2-way, LRU, 2-cycle hit. The L1 caches are private to each core	

L2 cache	Per-block interleaving, shared, distributed, 64 B line-size, perfect	
